# Ultrasound-guided rhomboid intercostal block for myofascial pain syndrome: a prospective clinical study

**DOI:** 10.55730/1300-0144.5517

**Published:** 2022-08-30

**Authors:** Selin GÜVEN KÖSE, Halil Cihan KÖSE, Serkan TULGAR, Ömer Taylan AKKAYA

**Affiliations:** 1 Department of Pain Medicine, Health Science University Gaziler Physical Therapy and Rehabilitation Training and Research Hospital, Ankara, Turkey; 2 Department of Pain Medicine, Health Science University Dışkapı Yıldırım Beyazıt Training and Research Hospital, Ankara, Turkey; 3 Department of Anesthesiology and Reanimation, Faculty of Medicine, Samsun University, Samsun Training and Research Hospital, Samsun, Turkey

**Keywords:** Myofascial pain syndrome, rhomboid intercostal block, chronic pain, ultrasound guided

## Abstract

**Background/aim:**

Myofascial pain syndrome (MPS) is a common chronic pain syndrome that may affect quality of life, daily living activities, and psychological status. Ultrasound (US)-guided rhomboid intercostal block (RIB) is a recently defined plane block and used for chronic pain such as postmastectomy syndrome and MPS. Our aim was to evaluate the efficacy of US-guided RIB for the management of pain, quality of life, physical disability, and patient satisfaction in MPS.

**Materials and methods:**

In this prospective study, between February and March 2021, a total of 30 patients who applied with the diagnosis of MPS, were included. The patients received US-guided RIB. Pain intensity was evaluated using a numerical rating scale (NRS) at pretreatment, and just after the intervention, at day 1, and 1, 2, 4, and 6 weeks after the intervention. At pretreatment and 6 weeks after treatment, Short Form-36 Health Survey (SF-36) for health-related quality of life, Neck Disability Index (NDI), and patient satisfaction were evaluated.

**Results:**

There was a statistically significant decrease in average NRS immediately after treatment, at day 1 and week 1,2,4, and 6 compared to the pretreatment (p < 0.0001). The average SF-36 scores advanced at 6 weeks after treatment. There was a statistically significant reduction in mean NDI scores throughout the follow-up period (p < 0.001).

**Conclusion:**

Our study demonstrated that RIB had improved neck function, physical and mental quality of life, and patient satisfaction in MPS. Therefore, we think US-guided RIB could be an alternative treatment modality in patients suffering from MPS.

## 1. Introduction

Myofascial pain syndrome (MPS) is a common chronic pain syndrome accompanied by sensory and motor symptoms as well as autonomic phenomena. It is characterized by the presence of myofascial trigger points defined as palpable, hyperirritable bands or nodules. Contractile activity of myofascial trigger points and nociceptor stimulation responding to pathological processes in the muscle fascial layer leads to myofascial pain [1]. The pain often affects patients’ quality of life, daily living activities, and psychological status severely. Clinical guidelines recommend analgesic drugs, physical therapy, and interventional procedures in the management of MPS [2].

With the increasing use of ultrasound technology in regional anesthesia and pain medicine, newly defined interfascial plane blocks (IFB) have become more popular. Ultrasound (US)-guided rhomboid intercostal block (RIB) is a thoracic plane block recently defined by Elsharkawy et al. in a patient presenting with multiple rib fractures [3]. RIB involves the injection of local anesthetic (LA) into the plane between rhomboid major and intercostal muscles. RIB leads to adequate postoperative analgesia when performed at T 5–6 level, with dermatomal coverage from T2 to T9 on both anterior and posterior hemithorax [4,5]. Later, successful application of RIB is reported in chronic pain such as postmastectomy pain syndrome and MPS [6,7]. The most noticeable advantages of this technique are its safety and sonoanatomic simplicity.

Currently, a variety of superficial injection techniques, including trigger point injections, LA injections, dry needling, and acupuncture are commonly used in MPS [8]. However, deep injections in the lumbar and thoracic areas have been reported to be more effective in MPS treatment compared with superficial injections [9–11]. Therefore, we hypothesized that, for individuals with MPS, the RIB would impact treatment outcome regarding pain intensity. The first and principle aim of this study was to evaluate the analgesic effectiveness of RIB. Moreover, we aimed to evaluate the efficacy of the RIB on quality of life, physical disability, and patient satisfaction in MPS.

## 2. Materials and methods

### 2.1. Study design

Local ethics committee approval (Dışkapı Yıldırım Beyazıt Training and Research Hospital with decision number 98/05-19.10.2020) was obtained for this prospective, observational study. Our study was designed and conducted in accordance with the ethical principles specified in the Helsinki Declaration. All participants were informed about the study, and written informed consent was obtained from all patients. This study was registered at ClinicalTrials.org PRS under registration no. NCT04751110. STROBE checklist was used to help design and conduct the study.

The inclusion criteria for the study were as follows: (1) be aged between 18 and 60 years; (2) experienced pain for at least 12 weeks in the thoracic paravertebral region between T2-T9; (3) presence of at least one active myofascial trigger point (MTrPs); and (4) pain with a numerical rating scales (NRS) score ≥3 lasts for at least three months. The active MTrP diagnosis was based on the fulfillment of all the following criteria: (1) palpable taut band; (2) presence of a hypersensitive tender spot within the taut band; (3) patient recognition of familiar referred pain symptoms upon compression of the nodule; and (4) painful limitation of full stretch range of motion (12).

The exclusion criteria for the study were as follows: (1) cervical radiculopathy, fibromyalgia, shoulder joint disease, chronic fatigue syndrome, rheumatic diseases, history of surgery or traumatic event, and malignancy as a cause of pain (2) use of anticoagulants; (3) history of bupivacaine or dexamethasone allergy; (4) history of injection for MPS within the last six months; (5) decline to participate; and (6) pregnancy.

### 2.2. Intervention

**Figure 1:**
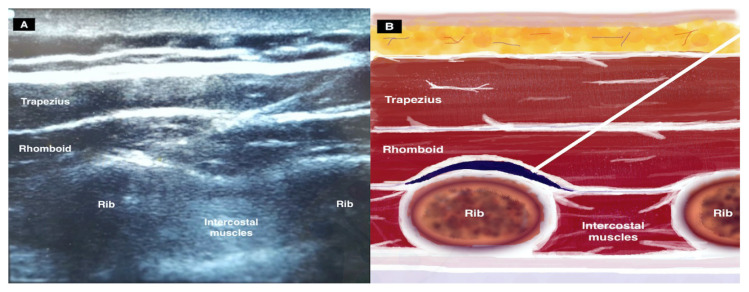
(a) Sonographic image and (b) schematic illustration showing the surrounding structures and needle position for rhomboid intercostal injection.

While the patient was positioned prone and the ipsilateral arm adducted across the chest, under aseptic conditions, a linear high-frequency ultrasound transducer was placed 2–3 cm medial of the medial border of the scapula on the sagittal plane at the T5–T6 level. Cutaneous and subcutaneous layers, the trapezius muscle, rhomboid major muscle, intercostal muscle, ribs, and pleura were visualized (Figure 1a). We inserted a 22-gauge needle into the tissue plane between the rhomboid major and intercostal muscles in a craniocaudal direction using an in-plane technique and injected 15 mL of 0.25% bupivacaine with 8 mg of dexamethasone into the fascial plane. Ultrasonography demonstrated the spread of LA in cranial and caudal directions under the rhomboid muscle (Figure 1b).

### 2.3. Outcome measurements

Descriptive data collected at baseline included age, gender, and body mass index (BMI). Patients were assessed at baseline, just after the intervention, at day 1, and 1, 2, 4, and 6 weeks after the intervention.

The NRS is a widely used scale to assess the severity of pain felt by a patient. The patient rates pain on a scale of 0 to 10, with 0 representing ‘no pain’ and 10 representing ‘worst pain’ imaginable. The NRS has been shown to have a strong correlation with descriptive scales, as well as high sensitivity and reliability [13]. Therefore, as a primary outcome measure, NRS assessment was applied during each visit.

The secondary outcome measures were quality of life, neck function, and patient satisfaction degree after treatment. The quality of life (QoL) was assessed via SF-36, which consists of 36 items encompassed in 8 dimensions: physical function, physical role, bodily pain, general health, vitality, social function, emotional role, and mental health and 2 summary values for Physical Component Summary (PCS) and Mental Component Summary (MCS) [14]. Subscale scores range from 0 to 100, with 100 as the most positive QoL in that area and 0 is the least; this scale has been validated in Turkish. The PCS and MCS of SF-36 were measured before treatment and 6 weeks after treatment.

Disability was measured by using the Neck Disability Index (NDI). The Turkish version of this scale was found valid and reliable. The questionnaire consists of 10 items in which each section is scored on a 0–5 rating scale, and the total score ranges from 0 to 50. Higher scores indicate greater disease severity [15]. NDI measurements were taken at baseline and at 6th week to document the difference from the baseline. Changes to overall satisfaction were assessed using a 5-point Likert scale [16] (1, very dissatisfied; 2, somewhat dissatisfied; 3, neutral; 4, somewhat satisfied; 5, very satisfied). No analgesic drugs were given to the patients during the study.

### 2.4. Statistical analysis

Sample size calculations were performed using G*Power software version 3.1.9.7 (Heinrich-Heine-Universität, Düsseldorf, Germany) according to our preliminary study data. In this study, we found the mean NRS score 2.5± 0.8 at 6 weeks. Using the results of this previous study and considering the NRS as a primary outcome, a sample size of 30 patients was determined to be necessary in order to detect a %20 difference, an α level of .05, and power of 80%.

Data were analyzed using the SPSS version 23.0 statistics program (IBM Corporation, Armonk, NY). Continuous quantitative data with normal distribution were presented as numbers, mean ± standard deviation, with abnormal distribution were presented as median (interquartile range). The compatibility of the variables to normal distribution was checked with a Kolmogorov–Smirnov test. The paired sample test was applied to parametric data for the statistical evaluation of repeated measurements. For abnormally distributed variables, intragroup distribution was compared using Friedman’s test. If present, within group comparisons of the differences were evaluated using the Bonferroni adjusted Wilcoxon signed ranks for post hoc analysis. P < 0.05 was considered statistically significant.

## 3. Results

**Figure 2:**
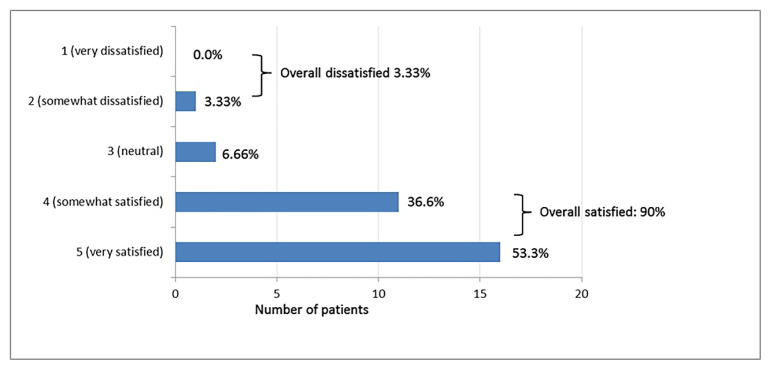
Patient-reported satisfaction with rhomboid intercostal block treatment protocol.

All 30 patients diagnosed with MPS completed the study. The demographic characteristics of patients are summarized in Table 1. There were no block-related adverse events, and no clinically apparent motor blockade was reported in any patients. The pretreatment NRS, NDI, and SF-36 scales were evaluated. The differences in the NRS scores of the patients were found to be statistically significant (**χ^2^ = 105.94**, p < 0.001) using a Friedman test. When binary time points were compared to find source of difference in NRS scores, there was a statistically significant difference immediately after treatment, at day 1 and week 1,2,4, and 6 compared to the pretreatment NRS (p < 0.001) (Table 2). There was also significant difference in NRS scores immediately after treatment and day 1 compared to week 6 (p < 0.001; p = 0.011). The average PCS of SF-36 advanced from 31.6 **±** 4.2 to 33.1 **±** 3.8 at 6 weeks after RIB. As for the MCS of SF-36, the average score increased from 35.1 **± 3.2 to 36.2 ±** 3.3 at 6 weeks after treatment. There was a statistically significant reduction in mean NDI scores throughout the follow-up period (p < 0.001). In total 53.3% of patients were very satisfied with pain management, 36.6% were satisfied, and 3.3% were not satisfied (Figure 2). Changes in the PCS and MCS of SF-36, and NDI are summarized in Table 3.

## 4. Discussion

In this study, we evaluated the clinical effect of US-guided RIB in patients with MPS. Our results showed that the severity of pain was significantly reduced after the block procedure for a 6-week follow-up period. Also, our study demonstrates that RIB improves the quality of life, disability, and patient satisfaction in the majority of the patients.

The ultrasound revolution has shown its effect on the practice of regional anesthesia and pain medicine. Nowadays, there is increasing interest and, therefore, new research studies in US-guided IFB. RIB is a recently demonstrated IFB as an alternative technique to the paravertebral and thoracic epidural block. The literature on RIB provides evidence that this technique can lead to effective and long-lasting perioperative analgesia in breast and thoracic surgeries [17,18]. Similarly, IFB such as erector spinae plane block and transversus abdominis plane block have been well-established techniques in perioperative pain control. Moreover, the rate of their use in chronic pain syndromes is gradually rising [19–21]. In 2011, Domingo et al. applied IFB with 10mL of 0.125% bupivacaine between the trapezius muscle and the levator scapulae in 25 patients with MPS. They reported that the mean VAS scores were reduced from 7.6 at pretreatment to 1.6 at 10 min after the procedure [22]. ESP block was successfully used in various cases having chronic pain syndrome [23,24]. Supportively, ESP block showed adequate pain control in a patient with right dorsal paravertebral pain from T4 to T11 due to MPS [25]. However, we only found two case reports regarding RIB in chronic pain management. In the first case, Piraccini et al. demonstrated that US-guided single shot RIB leads to effective relief in a patient with a tout band along the left paravertebral region and tender points. [6]. The second case was the successful application of RIB in a patient suffering chronic pain in the left dorsal hemithorax from T2 to T7 due to MPS [7]. Encouraged by the aforementioned cases in MPS and chronic pain syndromes treated with IFB, we performed RIB for MPS. At the end of the 6th week, we achieved improvement in patients with MPS. Similar to our study, Park et al. reported the successful administration of US-guided interfascial injection on MPS of the gastrocnemius. They injected 10 mL of 0.2% lidocaine into the gastrocnemius interfascial space in 20 patients with MPS. They reported that mean NRSs were significantly lower immediately after treatment, weeks 1, 2, and 4 than pretreatment. Also, mean PCS and MCS were significantly higher at four weeks’ posttreatment than pretreatment [26].

Fascia opens the possible keys for understanding the etiopathogenesis of pain syndromes such as chronic low back pain, myofascial pain syndrome, and fibromyalgia [27,28]. Several investigations indicate that fascia is richly innervated by both A- and C-fibers and contains free nerve endings, including Ruffini and Pacinian corpuscles. Also, nociceptive free nerve endings of muscles contain substance P and calcitonin gene-related peptide. These neuropeptides abundantly localize in the muscle fascia and contribute to the development of myofascial pain. Taken together, the literature has highlighted the role of the fascia in the occurrence of pain. Therefore, performing IFB with local anesthetics may control pain by reducing the ectopic discharges in nerve endings of the fascia [29–31].

Fascia has a dynamic structure regarding its mechanical and physiological properties. The autonomous contractile elements it has and the movement of the musculotendinous structures attached to it explain the dynamic properties of the fascia. [32]. Performing injections into interfascial planes, which act as a potential space, leads to a wide dermatomal distribution of LA regarding its dynamic structure. Deep fascial planes are transmission routes surrounding the musculoskeletal system [33]. The rhomboid intercostal plane extends to the erector spinae muscles medially and the serratus anterior muscle laterally. In a cadaveric examination, Elsharkawy et al. demonstrated the extent of contrast spread with RIB applied at the T6–7 level. They showed injectate spread to the lateral cutaneous branches of the thoracic nerves between T3 and T9, as well as posterior primary rami, between the intercostal muscles and deep to the serratus anterior muscle [34]. This excessive distribution characteristic of RIB may explain the pain relief mechanism in patients with thoracic paravertebral pain.

In MPS, sensitization of polymodal type receptors (PMR) plays an important role in the development of the MTrPs. Superficial approaches such as dry needling, trigger point injections, and acupuncture provide analgesia by activating cutaneous PMR [35]. Stimulating deeper tissues as in IFB activates the PMR in not only cutaneous tissue but also muscle and fascia [36]. The afferent nociceptive neurons located in the deeper muscle and fascia have a more crucial role in the transmission of analgesic stimulus than cutaneous afferents. Several studies compared the effect of LA injections applied at varying depths. Okmen et al. showed that US-guided deep injection of the rhomboid major muscle was more effective than the superficial injection of the trapezius muscle for pain, disability, and quality of life scores in patients with MPS [9]. In a randomized controlled study, deep acupuncture was found to result in a significantly better therapeutic effect compared to superficial acupuncture in patients with lumbar MPS [10]. As a result, RIB may have been accomplished under US guidance for MPS by providing more efficacious stimulation and up-regulation of the PMR in the track of the needle through the skin, subcutaneous tissue, trapezius and rhomboid muscle, and fascias.

Some limitations of this study should be considered. First, it had a short follow-up period; the effects were evaluated in only 6 weeks. Second, although our study could be criticized for the lack of a sham group, we considered that it is unethical to apply RIB in a placebo group with saline or intentionally puncture the fascia of muscles. However, this study could provide data for a sample size calculation for future randomized controlled trials. Third, NRS, Visual Analogue Scale, McGill pain questionnaire, and Pain Rating Scale are widely used scales for assessing pain intensity, however, we only used the NRS score as a primary outcome measure.

In conclusion, this observational clinical study demonstrated that the US-guided RIB could be an alternative treatment modality in patients suffering from myofascial pain. Furthermore, our study showed that RIB had improved neck function, physical and mental quality of life, and patient satisfaction among patients with MPS. We suggest that RIB may gain an essential place in the treatment algorithm of MPS. However, further clinical studies are required to compare its effectiveness and safety to conventional techniques performed in MPS.

## Figures and Tables

**Table 1:** Baseline sociodemographic characteristics of the patients.

Age (year)	49.03 ± 11.3
Body Mass Index (kg/m^2^)	28.4 ± 4.2
Symptom duration (year)	2.1 (1–5)
Gender (M/F)	8/22
Affected body side (right/left)	20/10

The values are presented as mean ± standard deviation, median (minimum-maximum) and numbers of patients.

**Table 2:** Summary of pain numeric rating scale scores (0–10).

NRS	NRS scores median	NRS min-max	p
Pretreatment	5 (4–6)	4–7	
Immediately after treatment	1 (0.25–1)	0–3	<0.001
Day 1	1 (1–2)	0–3	<0.001
Week 1	2 (1–2)	0–3	<0.001
Week 2	1 (1–2)	0–3	<0.001
Week 4	2 (1–2)	0–3	<0.001
Week 6	2 (2–3)	0–3	<0.001

Data are expressed as median (percentiles 25–75) and min-max boundaries.

NRS, numeric rating scale.

P values indicate Bonferroni adjusted Wilcoxon signed-ranks test statistical values.

P values comparing NRS between pretreatment vs. immediately after treatment, 1 day, 1, 2, 4, 6 weeks. P values were italicized and written in bold to represent statistical significance.

**Table 3:** Outcomes of the rhomboid intercostal block.

	Pretreatment	6. week	p
PCS of SF-36	31.6 ± 4.2	33.1 ± 3.8	0.152
MCS of SF-36	35.1 ± 3.2	36.2 ± 3.3	0.188
NDI	20.96 ± 4.24	11.60 ± 2.98	<0.001

Values are presented as numbers or mean ± standard deviation.

PCS, physical component summary score of the Short Form-36 health survey (SF-36);

MCS, mental component summary score of the SF-36; NDI, neck disability index

Statistically significant at the p < 0.05 level.

P values were italicized and p values that are written in bold represent statistical significance.
